# Is it safe to initiate the minimally invasive aortic valve replacement technique in an institution with experience in cardiovascular surgery?

**DOI:** 10.1186/1749-8090-10-S1-A172

**Published:** 2015-12-16

**Authors:** Rafael Meza, Alejandro Escobar, Gloria Franco, Alejandra Echeverri, Jose David Serna

**Affiliations:** 1 Department of Cardiovascular Surgery, Department of Epidemiology, Centro Cardiovascular Somer Incare, Rionegro, Colombia

## Background/Introduction

The implementation of new surgical techniques in an institution requires the suitable training of an interdisciplinary group (surgeons, anesthesiologists, nurses, doctors, ICU); the initial results, known as learning curve, are those that define the successful continuity of said techniques in the institutions, therefore they are of utmost importance.

## Aims/Objectives

Compare the pertaining patients to the learning curve group with the rest, both subjected to aortic valve replacement by the minimally invasive technique (MICS) conducted by the same group of cardiovascular surgeons, evaluating surgery time, postoperative complications, hospital stay and mortality.

## Method

83 consecutive cases were studied, with an age of 62.54 ± 12.60 years between 30 and 84 years, of them 70 (84.30%) were given an implanted Biological prosthesis and 13 (15.70%) a mechanical prosthesis, defined the learning curve as the first 25 procedures (Learning Curve) and compared with the 58 remaining procedures (The Rest).

## Results

The cross clamp time was 71.84 ± 10.85 min in the learning curve and 79.86 ± 21.079 in the Rest p = 0.076 and the perfusion time 83.32 ± 12.95 min in the learning curve and 95.02 ± 26.21 min in the rest p = 0.057 without significant differences. The main complications are shown in table 1 without significant differences

**Table 1:** 

	Complication Learning Curve Rest				
	
		n %		n %	p
Arrhythmias	2	8.00%	9	15.50%	0.354
Mediastinitis	0	0.00%	2	3.40%	0.347
SSI	1	4.00%	1	1.70%	0.535
Reintervention due to bleeding	1	4.00%	3	5.20%	0.819
Death<30 days	0	0.00%	2	3.40%	0.347

Hospital stay 6.72 ± 4.04 days Vs 7.16 ± 5.071 days p = 0.705 did not present statistically significant differences.

## Discussion/Conclusion

The learning curve in aortic valve replacement conducted by MICS can be initiated safely with a low morbimortality.

